# Inequalities in population health loss by multiple deprivation: COVID-19 and pre-pandemic all-cause disability-adjusted life years (DALYs) in Scotland

**DOI:** 10.1186/s12939-021-01547-7

**Published:** 2021-09-26

**Authors:** Grant M. A. Wyper, Eilidh Fletcher, Ian Grant, Oliver Harding, Maria Teresa de Haro Moro, Diane L. Stockton, Gerry McCartney

**Affiliations:** 1 Place and Wellbeing Directorate, Public Health Scotland, Glasgow, Scotland; 2 Data Driven Innovation Directorate, Public Health Scotland, Edinburgh, Scotland; 3 Directorate of Public Health, NHS Forth Valley, Stirling, Scotland; 4 Clinical and Protecting Health Directorate, Public Health Scotland, Edinburgh, Scotland

**Keywords:** Inequalities, COVID-19, Coronavirus, Disability-adjusted life year, DALY, Burden of disease, Population health, Scottish burden of disease

## Abstract

**Background:**

COVID-19 has caused almost unprecedented change across health, education, the economy and social interaction. It is widely understood that the existing mechanisms which shape health inequalities have resulted in COVID-19 outcomes following this same, familiar, pattern. Our aim was to estimate inequalities in the population health impact of COVID-19 in Scotland, measured by disability-adjusted life years (DALYs) in 2020. Our secondary aim was to scale overall, and inequalities in, COVID-19 DALYs against the level of pre-pandemic inequalities in all-cause DALYs, derived from the Scottish Burden of Disease (SBoD) study.

**Methods:**

National deaths and daily case data were input into the European Burden of Disease Network consensus model to estimate DALYs. Total Years of Life Lost (YLL) were estimated for each area-based deprivation quintile of the Scottish population. Years Lived with Disability were proportionately distributed to deprivation quintiles, based on YLL estimates. Inequalities were measured by: the range, Relative Index of Inequality (RII), Slope Index of Inequality (SII), and attributable DALYs were estimated by using the least deprived quintile as a reference.

**Results:**

Marked inequalities were observed across several measures. The SII range was 2048 to 2289 COVID-19 DALYs per 100,000 population. The rate in the most deprived areas was around 58% higher than the mean population rate (RII = 1.16), with 40% of COVID-19 DALYs attributed to differences in area-based deprivation. Overall DALYs due to COVID-19 ranged from 7 to 20% of the annual pre-pandemic impact of inequalities in health loss combined across all causes.

**Conclusion:**

The substantial population health impact of COVID-19 in Scotland was not shared equally across areas experiencing different levels of deprivation. The extent of inequality due to COVID-19 was similar to averting all annual DALYs due to diabetes. In the wider context of population health loss, overall ill-health and mortality due to COVID-19 was, at most, a fifth of the annual population health loss due to inequalities in multiple deprivation. Implementing effective policy interventions to reduce health inequalities must be at the forefront of plans to recover and improve population health.

**Supplementary Information:**

The online version contains supplementary material available at 10.1186/s12939-021-01547-7.

## Background

COVID-19 has caused almost unprecedented change across health, education, the economy and in social interaction. In Scotland in 2020, COVID-19 has been estimated to have had the second largest population health impact across all individual causes of disease and injury, with 98% of its impact attributed to premature mortality [1]. Prior to the pandemic, we knew that inequalities in health have persisted, even during periods of larger gains in population health [2]. It is widely understood that the existing mechanisms which shape health inequalities have resulted in COVID-19 outcomes following this same, familiar, pattern [3, 4].

As a response to mitigate the potential for COVID-19 impacting those that were already vulnerable to adverse COVID-19 outcomes, the Scottish Government developed shielding guidance towards the end of March 2020 [5]. The guidance mainly involved prolonged indoor social isolation, designed to protect those that were likely at greatest risk of COVID-19 infection and mortality. This involved shielding based on an age criteria, given that elderly people were at the greatest threat, and shielding of those with pre-existing underlying health conditions [6].

Published statistics have since underlined that not only was COVID-19 infection more common in Scotland’s most deprived areas, but the most deprived areas in Scotland tended to have higher rates of COVID-19 mortality [7, 8]. Commentators have since highlighted how the impact of COVID-19 infection has been magnified through a legacy of systemic and intersectional inequality, giving rise to a syndemic in some countries [9, 10].

The extent of health inequalities for individual health conditions in Scotland is well known. Causes such as: drug use disorders; liver cirrhosis; chronic obstructive pulmonary disease; and, suicide, exhibit the largest inequalities in health outcomes [11, 12]. On the other hand, there are health conditions where there are very little, to no, inequalities, such is the case for: sensory conditions; headaches; and some musculoskeletal conditions [13]. Although inequalities in COVID-19 outcomes have been reported, they have not yet been framed in a way that is comparable with all other common causes of morbidity and mortality, which risks biasing how they are interpreted within a wider context. Through using disability-adjusted life years (DALYs) to proportionately weight the impact of ill-health from morbidity, and premature mortality, due to individual causes of disease and injury, we can comprehensively, and comparably, examine the extent of COVID-19 inequalities with all other causes of health loss [14].

The aim of this paper was to estimate inequalities in COVID-19 population health loss in Scotland, measured by DALYs in 2020. Our secondary aim was to scale overall, and inequalities in, COVID-19 DALYs against the level of pre-pandemic inequalities in all-cause DALYs, derived from the Scottish Burden of Disease (SBoD) study.

## Methods

### Study population and period

The setting of this study was Scotland, which has a residential population of approximately 5.5 million [15]. The study time period for assessing COVID-19 DALYs was calendar year 2020. Inequalities in pre-pandemic health loss was characterised using the most recently available DALY estimates for calendar year 2018.

### Data

Individual-level data on provisional death registrations were sourced from National Records of Scotland (NRS) [16]. COVID-19 deaths were defined using the ICD-10 codes U07.1 or U07.2, based on guidance from the World Health Organization [17]. Deaths were included in this study if the date of death was during 2020, with the first death occurring in mid-March 2020. The dataset was generated on 25th February 2021, to allow additional time to capture the later registrations of some deaths occurring towards the end of 2020. Each individual death record had a postcode assigned. We matched each individual death to the area-based Scottish Index of Multiple Deprivation (SIMD) 2020 reference file, which is an area-based proxy of socioeconomic status, to group deaths based on socioeconomic quintile [18]. SIMD uses seven domains to examine the extent of area deprivation: income; employment; education; health; access to services; crime; and, housing. The inclusion of a health domain introduces a circular logic when assessing health outcomes, but previous studies have evidenced that it doesn’t make any practical difference.

Data on the number of Years Lived with Disability (YLD) directly due to COVID-19 infection were sourced through the SBoD study [1]. The SBoD study is a national, and local, population health surveillance system which monitors how diseases and injuries prevent the Scottish population from living longer lives in better health, and it’s methods are described in further detail elsewhere [1, 19, 20]. Mid-year population estimates by deprivation quintile were sourced from NRS [15]. The most recent pre-pandemic estimates of the number of DALYs due to all-causes in 2018 by deprivation quintile were sourced from the SBoD study [21].

### Analyses

The estimation of DALYs directly due to COVID-19 were based on the consensus model and methods outlined by the European Burden of Disease Network and the European Centre for Disease Prevention and Control [22]. We used two different definitions of mortality, which were used to estimate the upper and lower limits of DALYs. These were based on COVID-19 as the underlying cause of death (COVID-19 cause-specific), with the other definition being based on COVID-19 as an underlying or contributing cause of death (COVID-19 related). A small number of deaths that did not have a postcode, and as such did not match to a deprivation quintile (*N* = 17 (0.2%) for COVID-19 related deaths). These were treated as missing data, which we proportionately redistributed to deprivation quintiles based on the observed proportions of deaths by age group and deprivation quintile [23].

Mortality counts were aggregated by five-year age-group to calculate Years of Life Lost to premature mortality (YLL), with the under-5 year age-group split into under 1 year and 1 to 4 years, and the upper open-ended age-group set at 95 years and above. YLL was estimated by multiplying the number of deaths in each age-group by the age conditional life expectancy defined by the Global Burden of Disease 2019 reference life table [24]. The units of analyses were deprivation quintiles, which represent ordered fifths of the population, grouped on the basis of their overall SIMD score. The Scottish national estimate of YLD was proportionately redistributed to deprivation quintiles, based on our observed distribution of YLL across deprivation quintiles. YLD and YLL were summed to estimate DALYs.

Rates of COVID-19 DALYs per 100,000 population were estimated for each deprivation quintile. We estimated inequalities using several measures. Absolute and relative range differences in rates were estimated between the most and least deprived quintiles. Additionally, inequalities were measured using the Relative Index of Inequality (RII) and Slope Index of Inequality (SII). This involved fitting a linear regression to the ranked area deprivation quintiles and the DALY values. The RII was estimated by dividing the SII rate of DALYs by the overall Scottish rate of DALYs [25]. DALYs attributable to area deprivation were estimated by using the least deprived quintile as the reference group and summing all DALYs in excess of this reference across the other four deprivation quintiles, and are presented as numbers and in percentage terms [26]. Overall COVID-19 DALYs were described alongside inequalities in pre-pandemic all-cause DALYs, based on comparisons between absolute and relative measures of inequalities, and attributable DALYs. Additionally, COVID-19 DALYs were scaled against the extent of all-cause DALYs due to inequality in area deprivation.

All results were presented as a range (where appropriate), using a sensitivity based on YLL estimates using COVID-19 cause-specific, and related, deaths. All COVID-19 estimates presented were for calendar year 2020, in Scotland.

## Results

### Inequalities in the population impact of COVID-19

**Fig. 1:**
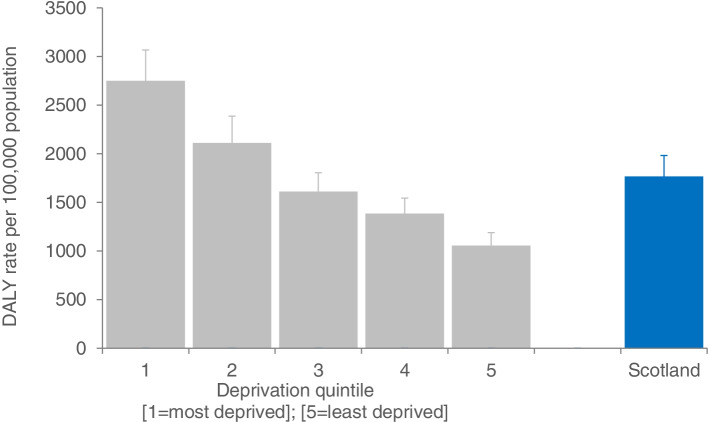
Disability-adjusted life years directly due to COVID-19, by area deprivation, Scotland, 2020. DALYs denote ‘disability-adjusted life years’; bars represent the lower rate limit for COVID-19 DALYs; extended error bars represent the range between the lower and upper rate limit; overall Scottish rate is given in blue

The overall rate of COVID-19 DALYs (DALYs (N) = 96,519 to 108,243) in Scotland, 2020, was estimated to be between 1767 and 1981 per 100,000 population (Fig. 1). The rate in the least deprived quintile was between 1054 to 1189, and the rate in the most deprived quintile ranged from 2748 to 3067.

### Comparisons between COVID-19 and all-cause inequalities

There were marked differences in the rate of COVID-19 DALYs by area deprivation, confirmed across all measures of inequality (Table 1). The absolute difference in DALYs between the most and least deprived areas was between 1694 and 1878 per 100,000 population. The relative difference in DALYs between the most and least deprived areas was 2.6, indicating that DALYs were 2.6 times higher in our most, compared to least, deprived areas. The RII was 1.16, which when multiplied by 0.5 and expressed as a percentage means that the rate in the most deprived areas was 58% higher than the mean rate of the population. The difference between the most and least deprived areas, measured by SII, ranged from 2048 to 2289 DALYs per 100,000. DALYs attributable to differences in area deprivation accounted for 40% of total COVID-19 DALYs (DALYs (N) = 38,925 to 43,284). Thus, inequalities in COVID-19 DALYs were higher than observed for pre-pandemic all-causes, as observed through differences using the relative, and percentage measures. COVID-19 has thus increased overall inequalities in DALYs across all measures of inequality.

**Table 1:** Inequalities in COVID-19 disability-adjusted life years (DALYs), Scotland, 2020

**Measure of inequality**	**COVID-19**		**Pre-pandemic all-cause**
	**DALYs lower limit**	**DALYs upper limit**	
Absolute rate difference (/100,000 population)	1694	1878	20,667
Relative rate difference	2.61	2.58	1.97
Relative Index of Inequality	1.16	1.16	0.84
Slope Index of Inequality (/100,000 population)	2048	2289	26,241
Attributable risk (%)	40%	40%	31%
Attributable risk (N)	38,925	43,284	535,298

‘DALYs’ denote disability-adjusted life years; attributable risk is the theoretical percentage/number of DALYs that could have been averted if all socioeconomic groups had the same observed DALY rate as the least deprived group

Comparing the rate of overall COVID-19 DALYs with inequalities in pre-pandemic all-cause DALYs yielded several insights. The overall rate of COVID-19 DALYs was 9 to 10% of the rate of DALYs for pre-pandemic inequality across all-causes, measured through the absolute rate difference. When the SII was used to measure the rate of absolute inequality, this dropped to 7 to 8%. However, when comparisons were made between overall COVID-19 DALYs and the number of DALYs attributable to area deprivation, it increased to 18 to 20%.

## Discussion

### Summary

COVID-19 has had a substantial impact on the population health in Scotland in 2020, however the impact was not shared equally across areas experiencing different levels of deprivation. This result was confirmed across all measures of inequality. There was a two-and-a-half fold difference in the rate of COVID-19 DALYs between the most and least deprived areas of Scotland. Generally, relative inequalities due to COVID-19 were greater than the inequalities in the overall all-cause pre-pandemic burden of disease. This is consistent with the idea that the population health impact of COVID-19 infection is largely due to mortality, as inequalities in mortality outcomes have been previously shown to be much larger than inequalities in ill-health due to morbidity [13]. Furthermore, if the rate of COVID-19 DALYs observed in Scotland’s least deprived areas had been observed in all other areas, 40% of DALYs could potentially have been averted. In terms of absolute impact, this would be equivalent to averting all DALYs due to diabetes in 2018 [1]. However, the overall impact of COVID-19 in Scotland in 2020 was, at most, a fifth of the annual pre-pandemic impact of inequalities in all-cause health loss, a result which ranged from 7 to 20%, depending on which measure of inequality was used.

Our findings, support the idea that there would be a high proportion of DALYs that are attributable to prior exposure to metabolic (such as obesity) and behavioural (such as tobacco smoking) risks [6, 27–29]. The prevalence of such risks factors are socially patterned, as are our findings, which underscore that preventative action to improve, and reduce inequalities in, population health is a matter of urgency, particularly given the recent slowdown of gains, and widening of inequalities, in mortality [30, 31].

### Strengths and limitations

In the early months of COVID-19 pandemic, there was potential for deaths not to be certified as COVID-19, particularly in the absence of a positive test. It is difficult to quantify any bias related to under- or over-recording of COVID-19 deaths. Therefore, we have presented our estimates as a range, to incorporate the main factors which are likely to have the largest impact on our estimates.

Our analyses assumed that the distribution of YLD by area deprivation was the same as the proportional distribution of YLL. We believe this to be a rational choice, given that total YLD is estimated to be around 2% of DALYs, and that the pattern of cases which have been observed have indicated similar patterns by deprivation [32]. Area deprivation is however known to underestimate the true inequalities experienced by socioeconomic position because only a proportion of the most deprived individuals live in deprived areas (and vice versa). Thus, the inequalities and attributable fractions for individual socioeconomic position would be likely to be higher than those reported here [33].

A major strength of our study is that DALYs capture the full impact of ill-health and mortality, across all causes of disease and injury. For example, comparisons that are made only using mortality data are useful, but ignore the consequences that people suffer due to living with health conditions. This means that our comparisons are among the most equitable, and comprehensive, that can be made.

When estimating YLL we used an aspirational approach, which assigns higher values of life expectancy than those currently achieved in Scotland [34]. Not only is it important for assessing the burden of disease overall in Scotland, but retaining this approach is important from an inequalities perspective. This ensures that we highlight the extent of inequalities relative to what could be achieved, rather than just what has recently been estimated for Scotland.

### Implications for policy and research

Progress of the COVID-19 vaccination programme (as at early spring 2021) has indicated high uptake, across all area-based deprivation groups. For elderly groups, uptake was lower in more deprived areas. There was a higher uptake of vaccination in the most deprived areas for people aged 50 to 64, although this was likely reflective of a higher proportion of clinically vulnerably patients in these groups being invited first [35]. Continuation of efforts to reduce inequalities in uptake, particularly amongst the elderly, will be an important tool in preventing adverse outcomes overall, and inequalities by socioeconomic position.

Previous work has indicated the potential scale of several early pandemic mortality scenarios, relative to inequality-related death in Scotland [36]. Our findings are consistent with the main messages from this, and further provide an up-to-date assessment of the 2020 impact which indicates that although COVID-19 infection has had a major population impact, its impact was substantially lower than the impact of all-cause pre-pandemic health inequalities across all-cause morbidity and mortality. The rapid, and substantial, resources deployed to tackle COVID-19 has demonstrated the potential for investment to tackle major public health issues in a swift manner. Although the absolute impact on population health through socioeconomic inequalities is at least five-fold of the impact of direct COVID-19 impact in Scotland, the policy response to COVID-19 has undoubtedly averted and mitigated from a much worse scenario, including the further exacerbation of health inequalities. During the COVID-19 pandemic there has been an increased public interest in accessing and understanding population health information [37]. Framing population health issues relative to COVID-19 offer opportunities to retain, and improve, public- and policy-level empowerment over tackling public health issues which existed prior to COVID-19, and present major future challenges.

There are major current, and future, challenges related to the wider pandemic harms due to disruptions to vital services, worsening of individual, and collective, social and economic circumstances [3]. In addition to this, there will also be clinically-related harms from long COVID-19 and indirect harms from suffering from infection, such as increased vulnerability and frailty. Reports during 2020 have indicated major concerns across several areas of health, relating to the increasing occurrence, and severity, of disease [38–40]. The SBoD study will continue to monitor mortality and morbidity to assess how short- and longer-term changes are proportionately impacting population health.

## Conclusion

COVID-19 has had a substantial impact on the population health in Scotland in 2020, however the impact was not shared equally across areas experiencing different levels of deprivation. The extent of inequality in population health loss was proportionate to averting all annual DALYs due to diabetes. In the wider context of population health loss, overall ill-health and mortality due to COVID-19 was at most a fifth of the annual population health loss due to inequalities in multiple deprivation. Implementing effective policy interventions to reduce these unfair, and unjust, health inequalities due to COVID-19, and all other causes, must be at the forefront of plans to recover and improve population health. Pursuing this will not only improve the lives of people in Scotland, but will ensure increased preparedness against any direct or indirect harms from future disasters of epidemics [41, 42].

## Supplementary Information

**Additional file 1.**

## Data Availability

Summary data used to construct the figure and table in this article are available as supplementary material. The underlying data that support the findings of this study are available from the eDRIS team at Public Health Scotland (Tel: 0131 275 7333; Address: Farr Institute Scotland, Nine Edinburgh Bioquarter, Little France Road, Edinburgh, EH16 4UX), but restrictions apply to the availability of these data, which were used under permissions for the current study.

## Abbreviations

COVID-19: Coronavirus Disease 2019
DALYs: Disability-adjusted life years
ICD: International Classification of Diseases
N: Number
NRS: National Records of Scotland
RII: Relative Index of Inequality
SBoD: Scottish Burden of Disease
SII: Slope Index of Inequality
SIMD: Scottish Index of Multiple Deprivation
YLD: Years Lived with Disability
YLL: Years of Life Lost to premature mortality
